# Different aspects of hand grip performance associated with structural connectivity of distinct sensorimotor networks in chronic stroke

**DOI:** 10.14814/phy2.15659

**Published:** 2023-04-05

**Authors:** Christian Schranz, Shraddha Srivastava, Bryant A. Seamon, Barbara Marebwa, Leonardo Bonilha, Viswanathan Ramakrishnan, Janina Wilmskoetter, Richard R. Neptune, Steve A. Kautz, Na Jin Seo

**Affiliations:** ^1^ Department of Health Sciences and Research Medical University of South Carolina Charleston South Carolina USA; ^2^ Division of Physical Therapy, Department of Rehabilitation Sciences Medical University of South Carolina Charleston South Carolina USA; ^3^ Ralph H. Johnson VA Health Care System Charleston South Carolina USA; ^4^ Department of Neurology Medical University of South Carolina Charleston South Carolina USA; ^5^ Department of Neurology Emory University Atlanta Georgia USA; ^6^ Department of Public Health Sciences Medical University of South Carolina Charleston South Carolina USA; ^7^ Division of Speech‐Language Pathology, Department of Rehabilitation Sciences Medical University of South Carolina Charleston South Carolina USA; ^8^ Walker Department of Mechanical Engineering The University of Texas at Austin Austin Texas USA; ^9^ Division of Occupational Therapy, Department of Rehabilitation Sciences Medical University of South Carolina Charleston South Carolina USA

**Keywords:** brain networks, connectome, grip force, stroke, upper extremities

## Abstract

Knowledge regarding the neural origins of distinct upper extremity impairments may guide the choice of interventions to target neural structures responsible for specific impairments. This cross‐sectional pilot study investigated whether different brain networks explain distinct aspects of hand grip performance in stroke survivors. In 22 chronic stroke survivors, hand grip performance was characterized as grip strength, reaction, relaxation times, and control of grip force magnitude and direction. In addition, their brain structural connectomes were constructed from diffusion tensor MRI. Prominent networks were identified based on a two‐step factor analysis using the number of streamlines among brain regions relevant to sensorimotor function. We used regression models to estimate the predictive value of sensorimotor network connectivity for hand grip performance measures while controlling for stroke lesion volumes. Each hand grip performance measure correlated with the connectivity of distinct brain sensorimotor networks. These results suggest that different brain networks may be responsible for different aspects of hand grip performance, which leads to varying clinical presentations of upper extremity impairment following stroke. Understanding the brain network correlates for different hand grip performances may facilitate the development of personalized rehabilitation interventions to directly target the responsible brain network for specific impairments in individual patients, thus improving outcomes.

## Graphical abstract

Different aspects of hand grip performance may be associated with the structural connectivity of distinct sensorimotor networks in chronic stroke survivors. This knowledge may facilitate the development of personalized rehabilitation interventions to target the responsible brain network for specific motor impairments in individual patients, thus improving outcomes.

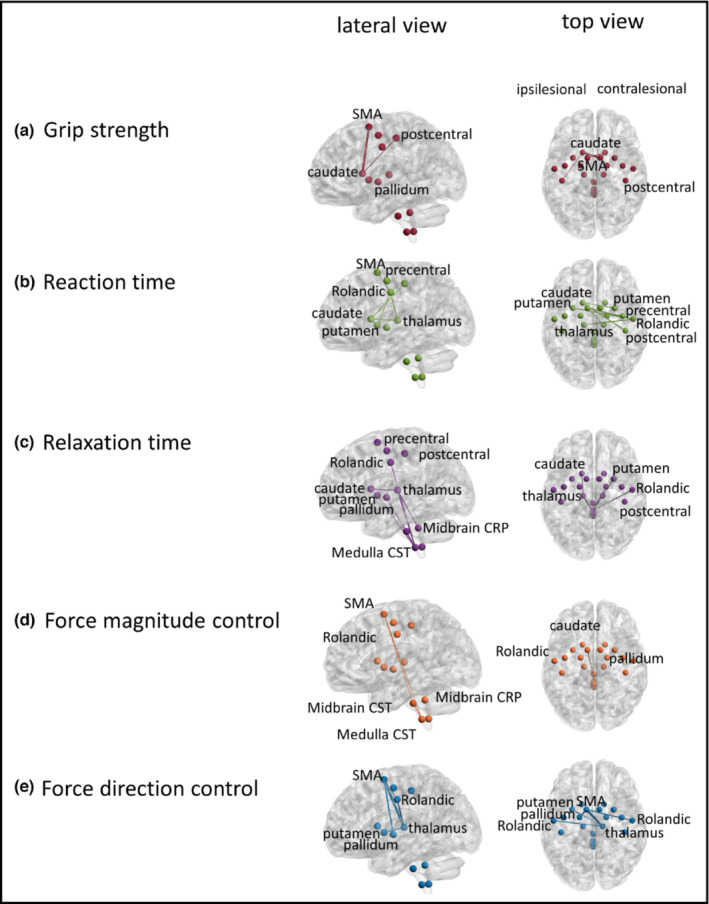

## 1 INTRODUCTION

Substantial heterogeneity exists among individuals in the presentation of motor impairment following a stroke. In the upper extremity, some stroke survivors experience more impairment in force production, while for others dexterous object manipulation may be the main challenge. This heterogeneity is due to variability in underlying neural impairment (Takeuchi & Izumi, 2013). Knowledge regarding the neural origins of distinct upper extremity impairments may guide the choice of rehabilitation interventions to target the neural structures responsible for the specific impairment.

For example, a study by Xu et al. (2017) showed that the overall upper limb function measured by the Fugl‐Meyer Assessment of Motor Recovery After Stroke—Upper Extremity (FMUE) was related to both grip strength and finger individuation. However, finger individuation strongly correlated with the cortical hand area lesion volume while grip strength did not. Similarly, hand dexterity has been attributed to corticospinal integrity, while synergy expression and muscle tone have been attributed to the reticulospinal pathway (McPherson et al., 2018). These studies suggest that the overall clinical function can be explained by detailed profiles of motor impairment, which are associated with distinct neural mechanisms allowing heterogeneity to be directly addressed.

However, current evidence is limited in terms of characterizing a detailed profile of motor impairment during grasping tasks and their individual neural correlates. As the next step, this pilot study represents a proof‐of‐concept demonstration of characterizing various aspects of hand grip performance and their neural correlates. We hypothesized that different aspects of hand grip performance (e.g., grip strength versus grip force direction; Seo et al., 2015) will be associated with distinct brain networks.

## 2 METHODS

### 2.1 Participants

Twenty‐two hemiparetic stroke survivors participated in this cross‐sectional pilot study. All participants were at least 6 months poststroke, with a mean time since the stroke of 5.3 ± 4.9 years (mean ± standard deviation, SD). The majority had an ischemic stroke while five participants had a hemorrhagic stroke. Participants were 61.6 ± 12.6 years old and were 14 males and 8 females. They had on average moderate upper limb impairment (FMUE = 43.9 ± 11.9). None had severe limb pain (visual analog scale ≥5) or severe sensory loss (National Institutes of Health Stroke Scale Sensory score = 2). All participants provided informed consent to the study protocol approved by the local Institutional Review Board. The procedures followed were in accordance with the ethical standards of the responsible institutional committee on human experimentation and with the Helsinki Declaration of 1975, as revised in 2008.

### 2.2 Hand grip performance measures

Grip strength is one of the most common measures of poststroke impairment and correlates with motor function (Stock et al., 2019). Reaction and relaxation times have been shown to be slowed poststroke and attributed to different neural mechanisms (Kamper et al., 2022; Motawar et al., 2012, 2016; Persson et al., 2020; Seo et al., 2009, 2011). In addition, proper control of grip force magnitude (Quaney et al., 2005) and direction (Seo et al., 2010, 2015) is essential to efficiently handle objects for task requirements and has been shown to be impaired and associated with upper limb function poststroke. These measures can be obtained simultaneously during a person's grip.

Participants were seated in front of a computer screen with the forearm rested on a table and the thumb and index finger on force sensors (Mini40, ATI Industrial Automation Inc, NC). Participants were instructed to grip the sensors with two parallel grip surfaces with their maximum force three times to obtain an average grip strength. In addition, participants were instructed to grip and relax 2 s later following visual cues on the computer screen generated by a custom LabVIEW program (National Instruments, Austin, TX). Participants gripped for a 4 N target force without visual feedback three times, after practicing with visual feedback. Three repetitions of each condition were performed the grip task with the nonparetic hand first, followed by the paretic hand. A custom LabVIEW program recorded the 3‐axis force data at 500 Hz.

**FIGURE 1:**
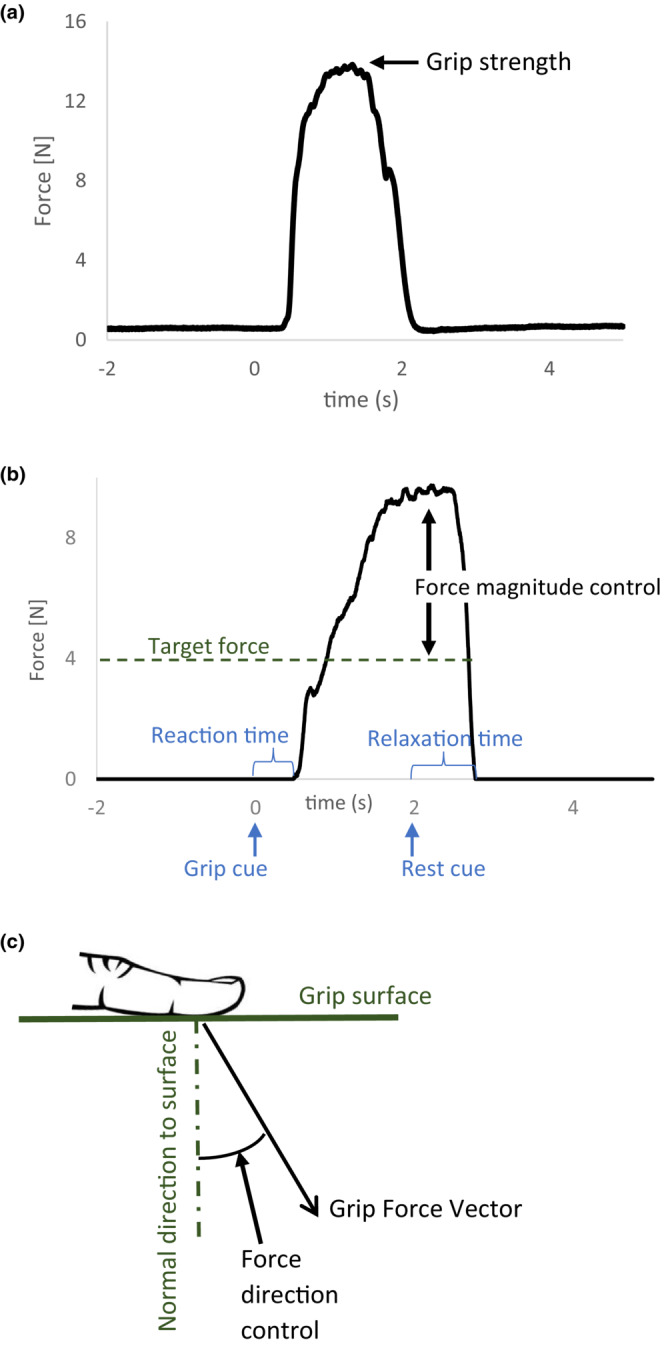
Illustration of the hand grip performance measures: grip strength (a), reaction time, relaxation time, and force magnitude control (b), and force direction control (c).

Individual grip performance measures were obtained using a custom program in MATLAB (MathWorks, Natick, MA). The force data were visually inspected for quality assurance. Strength was determined as the peak force observed during the maximum grip (Figure 1a). Reaction time was determined as the time duration from the grip cue to grip initiation defined as when grip force increased more than the baseline grip force by 3 SD (Hur et al., 2014; Seo et al., 2009). Relaxation time was determined as the time duration from the rest cue to grip termination defined as when grip force returned to the baseline force level within 3 SD (Seo et al., 2009). Force magnitude control was quantified as the absolute difference between the target force and mean grip force during grip (Figure 1b; Anderson et al., 2018; Quaney et al., 2005). Force direction control was quantified as the mean angular deviation of digit force from the direction normal to the grip surface during grip (Figure 1c; Seo et al., 2010, 2015). Repetitions were averaged. A ratio of the paretic value to the sum for both hands was computed to normalize the data and use in the statistical analysis.

### 2.3 Brain networks

While stroke damages specific regions, the functional consequences extend to brain networks disrupted by the lesions (Koch et al., 2021). Therefore, we used the connectome approach to quantify residual neural networks outside of the stroke lesion. All participants underwent MRI using a 3T Siemens Prisma scanner (Siemens Healthcare, Erlangen, Germany). A T1‐weighted magnetization‐prepared rapid acquisition gradient‐echo sequence (Brant‐Zawadzki et al., 1992) (TR:1900 ms, TE:2.26 ms, T1:900 ms, isotropic voxel size 1 mm × 1 mm × 1 mm), T2‐weighted FLAIR (TR: 9000 ms, TE:93 ms, T1: 2500 ms, voxel size: 0.9 mm × 0.9 mm × 0.9 mm), and diffusion tensor image (DTI) (TR: 6400 ms, TE: 96 ms, isotropic voxel size: 2.7 mm × 2.7 mm × 2.7 mm) were obtained.

**FIGURE 2:**
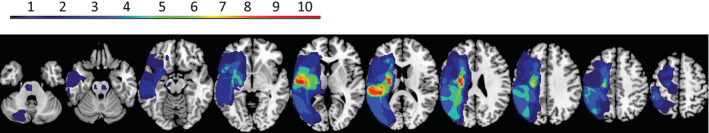
Lesion locations of participants: The affected hemisphere is shown on the left and the unaffected hemisphere on the right. The color indicates how many participants had a lesion at a spot.

Each participant's structural connectome was reconstructed using the previously published streamline approach (Bonilha, Nesland, Rorden, Fillmore, et al., 2014; Bonilha, Nesland, Rorden, & Fridriksson, 2014; Srivastava et al., 2022). First, brain lesions were manually traced on the T2 weighted FLAIR using MRIcron (Rorden & Brett, 2000). Additional regions of interest were manually traced for the corticoreticular pathway (CRP) in the reticular tract of medulla and midbrain; and for the corticospinal tract (CST) in the pyramid of the medulla and cerebral peduncle of the midbrain in DTI (Srivastava et al., 2022). The T2 was co‐registered with T1. The lesions were resliced using the transforms into the native T1 space, smoothed, and binarized. Uneven edges were eliminated using a 3‐mm full width at half‐maximum Gaussian kernel. A threshold of 0 was used for binarizing the lesion maps. A chimeric T1 was created by replacing lesioned areas with the mirrored image of the intact hemisphere (Bonilha, Nesland, Rorden, Fillmore, et al., 2014). The T1 was normalized onto the standard space with an enantiomorphic normalization approach (Nachev et al., 2008) in SPM12 (University College London, London, UK). An overlay of the lesion locations of participants in the standard space is shown in Figure 2. The T1 was segmented into probabilistic maps of white and gray matter using the unified segmentation normalization (Ashburner & Friston, 2005) in SPM12 with stroke lesion excluded. Gray matter was divided into the Atlas of Intrinsic Connectivity of Homotopic Areas (AICHA) (Joliot et al., 2015) and nonlinearly registered to the DTI space. The manually traced CRP and CST regions were merged with AICHA. The diffusion image was undistorted using Eddy (Andersson & Sotiropoulos, 2016). Pairwise probabilistic fiber tracking was performed using FMRIB Diffusion Toolbox probtrackX (Behrens et al., 2007) and its accelerated BEDPOST (Hernández et al., 2013) to obtain the number of streamlines connecting each pair of regions. The weighted connectivity pair between each pair of brain regions was obtained by averaging the probabilistic streamlines in both directions. The number of streamlines was corrected to account for unequal distances between regions and the size of gray matter regions using the distance between each region pairs, and the size of both regions of each region pairs (Bonilha et al., 2015; Bonilha, Nesland, Rorden, & Fridriksson, 2014; Gross, 2008).

The streamlined data among a reduced set of 20 brain regions from the AICHA atlas relevant to motor function were extracted. The cortical regions for the current analysis include the bilateral precentral, Rolandic, postcentral, and supplemental motor area (SMA) for their relevance in motor execution, sensory processing (Borich et al., 2015), and movement preparation/coordination (Welniarz et al., 2019). Subcortically, basal ganglia (putamen, caudate, pallidum) and thalamus were included for their relevance for motor performance and skill acquisition (Cataldi et al., 2021). These areas were included bilaterally, considering compensation by the contralesional hemisphere or interhemispheric inhibition (Ward et al., 2003). Additionally, CRP and CST were included for their relevance to muscle tone (Jang & Lee, 2019) and dexterous motor function, respectively (Zaaimi et al., 2012).

### 2.4 Statistical analysis

These streamlined data were analyzed with a two‐step factor analysis. Separately for each brain region, we performed a factor analysis of the streamlines between region *i* and the rest of the regions to produce factors for region *i*. The resulting factors were used for a second‐factor analysis to obtain final factors representing prominent brain networks.

Regression analyses were performed between each brain network as an independent variable and each grip performance as a dependent variable. To account for the confounding influence of the lesion volume, the percent lesion volume in each brain network was used as a covariate in each regression. None of the variance inflation factors of these regressions presented multicollinearity. Linear regression was used because there was no evidence of nonlinearity. Statistical analysis was carried out in IBM SPSS Statistics 27.

## 3 RESULTS

Participants' mean ± SD hand grip performance measures were as follows: grip strength 0.39 ± 0.20, reaction time 0.52 ± 0.11, relaxation time 0.54 ± 0.16, force magnitude control 0.50 ± 0.35 and force direction control 0.54 ± 0.11. They are expressed in the ratio of the paretic hand value to the sum for both hands. The factor analysis identified seven brain networks.

**FIGURE 3:**
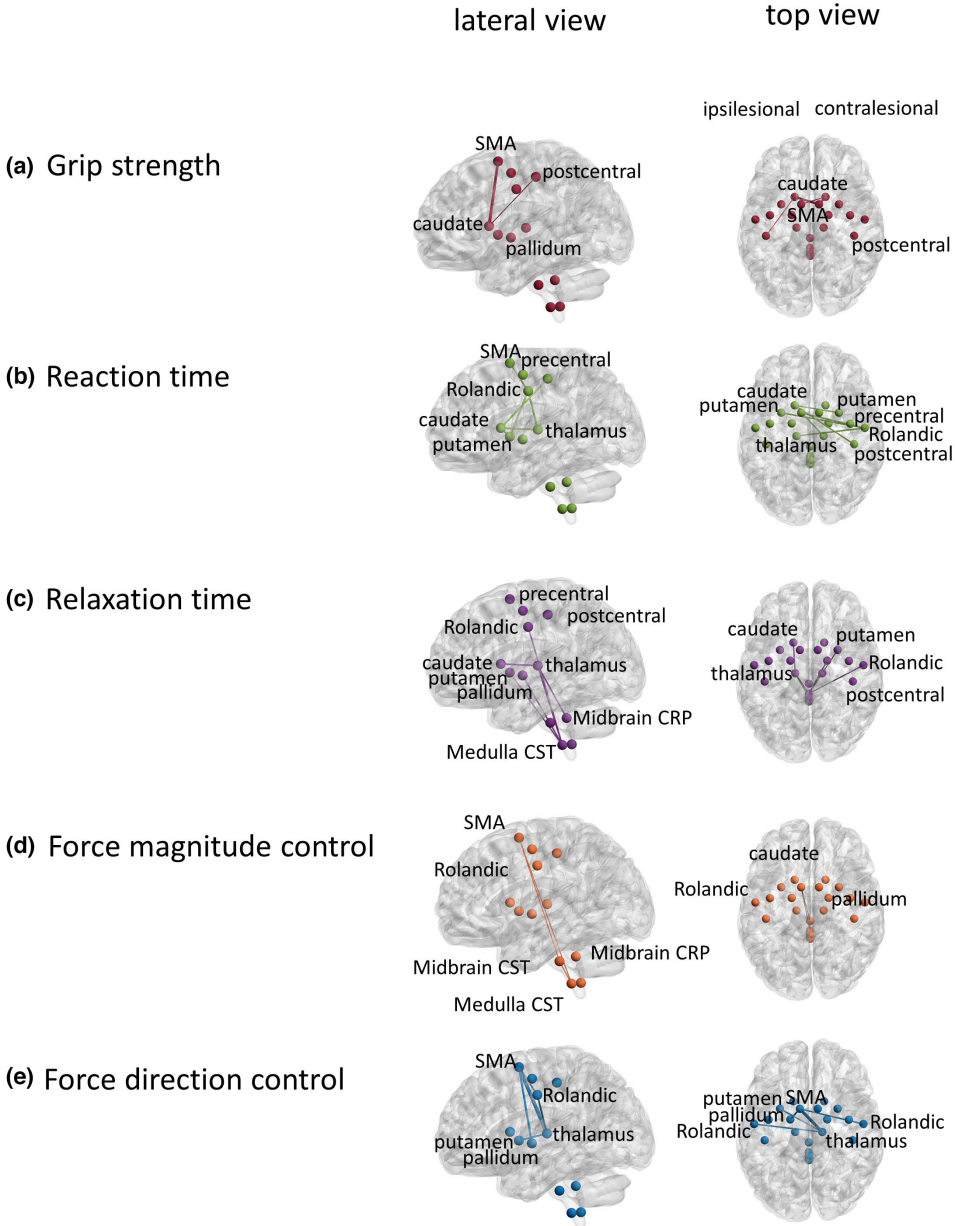
Brain networks related to hand grip performance measures: Lateral and top view of the five distinct brain networks that correlated best with each of the five grip performances: (a) maximum grip force, (b) reaction time, (c) relaxation time, (d) force magnitude control, and (e) force direction. In the top view, the lesioned hemisphere is shown on the left and the nonlesioned hemisphere on the right.

**FIGURE 4:**
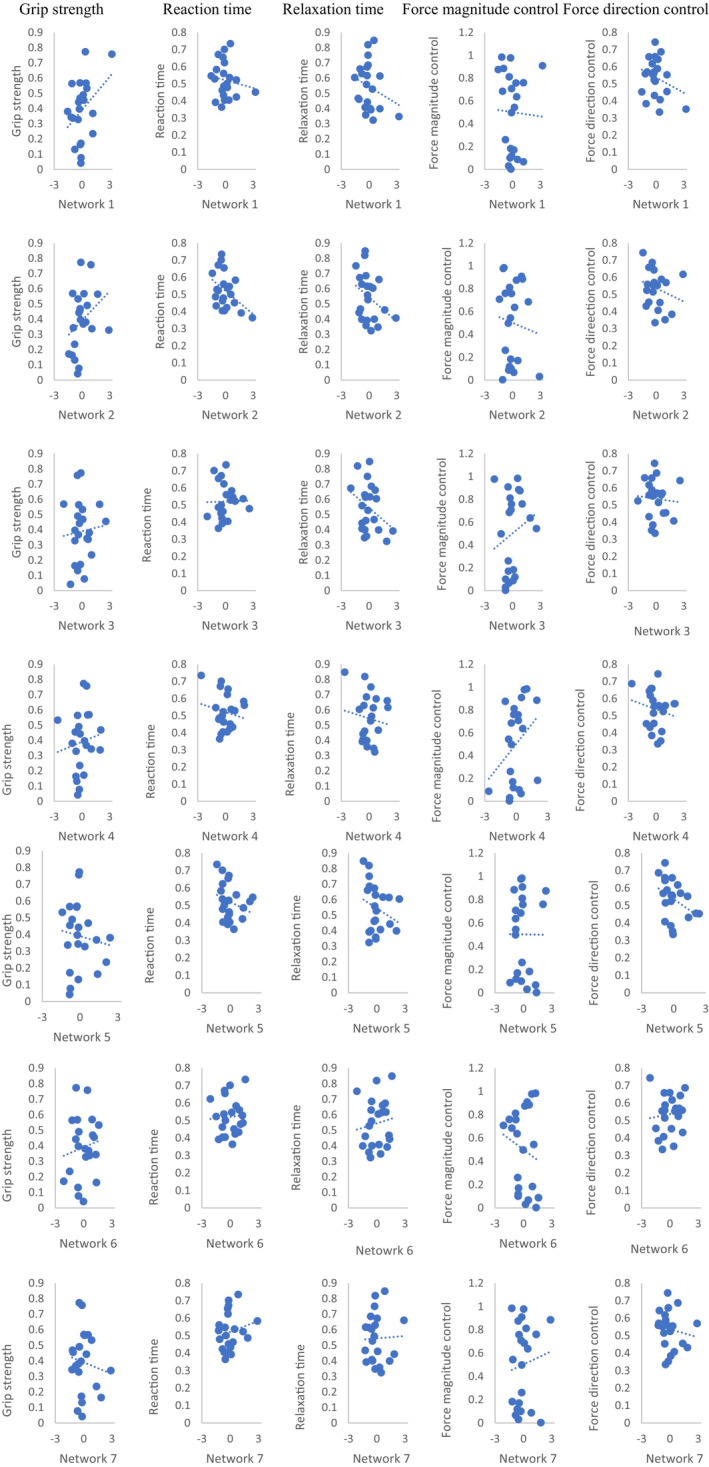
Correlation plots between each hand grip performance measure and brain network, not controlled for lesion volume.

Each of the five hand grip performance measures was best predicted by a different brain network (Table 1). Each network is described in Figure 3 with top 6 streamlines contributing to that network with the weight of at least 0.4. Grip strength was best explained by the brain network including streamlines between bilateral SMA and caudate and ipsilesional postcentral area (Figure 3a, R = 0.37). Reaction time was best explained by the network composed of interhemispheric streamlines among contralesional Rolandic and postcentral areas, ipsilesional SMA, and subcortical areas (Figure 3b, R = −0.39). Relaxation time was best explained by the network centered around the medulla CST, midbrain CRP, ipsilesional thalamus, contralesional Rolandic, and subcortical areas (Figure 3c, R = −0.36). Force magnitude control was best explained by the network consisting of ipsilesional SMA and medulla and midbrain CST (Figure 3d, R = 0.32). Force direction control was best explained by the network centered around ipsilesional SMA, contralesional thalamus, and bilateral Rolandic, pallidum, and putamen (Figure 3e, R = −0.39). Findings were consistent with versus without the lesion volume accounted for. None of the regressions reached significance at 5% level. Correlation plots of the relationship between brain networks and hand grip performance are presented in Figure 4.

**TABLE 1:** Correlation coefficients (*r*) between each hand grip performance measure and brain network, controlled for lesion volume. The slopes (beta) ± standard errors (SE) are also shown.

		Grip strength	Reaction time	Relaxation time	Force magnitude control	Force direction control
Network 1	*r*, beta ± SE	**0.37, 0.37** ± **0.043**	−0.14, −0.13 ± 0.023	−0.25, −0.22 ± 0.031	−0.03, −0.03 ± 0.080	−0.27, −0.25 ± 0.022
Network 2	*r*, beta ± SE	0.27, 0.28 ± 0.046	−**0.39,** −**0.41** ± **0.023**	−0.28, −0.29 ± 0.036	−0.04, −0.04 ± 0.086	−0.08, −0.08 ± 0.024
Network 3	*r*, beta ± SE	0.09, 0.09 ± 0.044	0.01, 0.01 ± 0.024	**−0.36,** −**0.35** ± **0.032**	0.19, 0.18 ± 0.079	−0.11, −0.10 ± 0.023
Network 4	*r*, beta ± SE	0.15, 0.15 ± 0.045	−0.20, −0.20 ± 0.023	−0.16, −0.16 ± 0.035	**0.32, 0.31** ± **0.077**	−0.26, −0.24 ± 0.022
Network 5	*r*, beta ± SE	−0.15, −0.15 ± 0.046	−0.24, −0.24 ± 0.023	−0.22, −0.22 ± 0.035	0.03, 0.03 ± 0.082	**−0.34,** −**0.31** ± **0.022**
Network 6	*r*, beta ± SE	0.09, 0.09 ± 0.046	0.11, 0.11 ± 0.024	0.18, 0.18 ± 0.035	−0.14, − 0.14 ± 0.082	0.24, 0.22 ± 0.023
Network 7	*r*, beta ± SE	−0.14, −0.14 ± 0.045	0.23, 0.22 ± 0.023	0.07, 0.06 ± 0.034	0.13, 0.13 ± 0.080	−0.11, −0.10 ± 0.023

Shades and bold values indicate the highest regression coefficient for each grip performance measure.

## 4 DISCUSSION

This is the first study to examine streamline‐based brain networks for various aspects of hand grip performance. Previous connectome research relied on a single motor function score (Xu et al., 2017). By contrast, the present study assessed multiple aspects of hand motor impairment and found that distinct networks are responsible for different aspects of hand motor performance. Thus, the present study characterizes heterogeneity in the presentation of upper extremity impairment with distinct neural network features. Exploring differing networks for different aspects of function in individuals with differing neurological insults may provide an interesting basis for future research investigating the neural correlates of ability.

In addition, this study examined brain networks for relaxation time and force direction control for the first time. Further, new networks found in the present study include interhemispheric connections for reaction time, brainstem pathways for relaxation time, and SMA/thalamus‐centered network for force direction control. These connections may represent interhemispheric inhibition necessary for task performance (Seo, 2013), corticospinal involvement for terminating muscle activity (Seo et al., 2009), and internal model for grip force control (White et al., 2013). For grip strength, force magnitude control, and reaction time, the networks included brain regions previously implicated from voxel‐based lesion symptom mapping (Liu et al., 2018; Weitnauer et al., 2021) and fMRI BOLD brain activity (Ward et al., 2003). They were basal ganglia, thalamus, and SMA. The basal ganglia have been shown to play a key role in grip force modulation (Pradhan et al., 2015). Internal regulation of the force increases the metabolic demands on the basal ganglia, especially on the pallidum. The caudate is responsible for the selection of the grip force that will be employed (Pradhan et al., 2015). The thalamus is important for the organization of movement and as a sensory hub of the brain (Hwang et al., 2017). Its projections relay nearly all afferent information to the cortex and mediate cortical communications (Hwang et al., 2017; Nikulin et al., 2008). Its anticipatory activity before the movement has been shown to correlate with shorter reaction times of upper extremity movements (Nikulin et al., 2008). The SMA is responsible for the internal model of force magnitude control (White et al., 2013). It receives the sensory information needed to predict the grip force needed for the task (White et al., 2013). Thus, the involvement of the SMA might be related to feedback motor control for desired force output.

This study has a significant implication for rehabilitation. First, the finding highlights the need to characterize various aspects of motor function to capture the heterogeneity in impairment following stroke and apply the most effective intervention for individual patients. Second, it provides an improved understanding of (Badran et al., 2020; Li et al., 2017; Palmer et al., 2018) brain networks associated with specific impairment. Third, this improved understanding might facilitate the development of personalized rehabilitation interventions such as brain stimulation (Badran et al., 2020; Li et al., 2017; Palmer et al., 2018) to directly target the brain network responsible for the specific impairment and enhance clinical outcomes in individual patients. Thus, this work extends the previous work of using brain networks for prognosis (Feng et al., 2015; Koch et al., 2021) by promoting the development of personalized rehabilitation treatment for individuals.

Results presented were based on a pilot study with a small sample size of 22. This exploratory study was performed primarily to evaluate the potential that brain networks for distinct motor performance aspects may be identified. The results of this study encourage future studies with a larger sample size for the definitive identification of the networks for varying demographic and clinical characteristics. Inclusion of neurotypical persons may also be considered to span a larger variability in structural networks. Future work should include connectome‐based lesion mapping (Weitnauer et al., 2021) to construct decision trees or target neuromodulation with larger datasets.

In conclusion, this pilot study provides insight that distinct brain networks may contribute to various aspects of hand functional impairment following stroke. The results implicated brain regions that have previously been associated with common hand function measures, in addition to revealing potential new networks. Pending larger studies for the definitive identification of networks and causal efficacy, this research may facilitate the development of personalized, network‐level intervention to improve function in stroke survivors.

## AUTHOR CONTRIBUTIONS

Christian Schranz performed the main data analysis including factor analyses, regressions, and interpretations, and drafted the manuscript. Shraddha Srivastava performed data collection and connectome construction. Bryant A. Seamon performed data collection and clinical assessments. Barbara Marebwa and Leonardo Bonilha contributed to the analysis of neuroimaging data and stroke lesion. Viswanathan Ramakrishnan and Janina Wilmskoetter contributed to statistical analysis. Richard R. Neptune, Steve Kautz, and Na Jin Seo contributed to the conception, design, and interpretation of the work. Na Jin Seo performed the analysis of the grip performances. All authors critically reviewed, revised, and approved the manuscript.

## FUNDING INFORMATION

This work was supported by the Rehabilitation Research & Development Service of the Department of Veteran Affairs through Grants 1I01RX001935 and I01RX003066, Senior Research Career Scientist award through Grant 1IK6RX003075, National Institute of General Medical Sciences P20GM109040, and National Institutes of Health/Eunice Kennedy Shriver National Institute of Child Health and Human Development R01HD094731.

## CONFLICT OF INTEREST STATEMENT

The authors report no competing interest.

## NEW AND NOTEWORTHY

Interhemispheric connections, brainstem pathways, and SMA/thalamus‐centered network were implicated for reaction time, relaxation time, and force direction control, respectively, in chronic stroke survivors. Different brain networks may be responsible for different aspects of hand grip performance and thus varying clinical presentations of upper extremity impairment following stroke. Understanding the brain network correlates for heterogeneous motor impairments may facilitate the development of personalized rehabilitation interventions.
